# The Uptake and Deconjugation of Androstenone Sulfate in the Adipose Tissue of the Boar

**DOI:** 10.3390/ani11113158

**Published:** 2021-11-05

**Authors:** Christine Bone, E. James Squires

**Affiliations:** Department of Animal Biosciences, University of Guelph, Guelph, ON N1G2W1, Canada; cbone@uoguelph.ca

**Keywords:** pig, boar taint, androstenone, androstenone sulfate, steroid uptake, deconjugation

## Abstract

**Simple Summary:**

Boar taint is a meat quality issue that results from the accumulation of androstenone in the adipose tissue. During steroid synthesis, steroids such as androstenone undergo sulfoconjugation, a process that involves the attachment of a sulfonate group to enhance polarity. Androstenone sulfate is more abundant in the plasma than free androstenone and is suspected to enzymatically regenerate free androstenone in peripheral tissues such as the fat to indirectly contribute to boar taint development. In this article, we identified a specific membrane transporter that is responsible for the uptake of androstenone sulfate into the fat and confirmed that androstenone sulfate can enzymatically regenerate free androstenone within the adipose tissue. We also identified a positive relationship between the quantity of free androstenone enzymatically produced from androstenone sulfate and fat androstenone concentrations in early maturing boars. These results suggest that the production of free androstenone from androstenone sulfate may contribute to the development of boar taint in early maturing animals.

**Abstract:**

Boars express high testicular levels of sulfotransferase enzymes, and consequently, the boar taint causing compound androstenone predominantly circulates as a steroid sulfate. Androstenone sulfate is suspected to function as a steroid reservoir that can be deconjugated to provide a source of free androstenone for accumulation. Therefore, the purpose of this study was to characterize the uptake and deconjugation of androstenone sulfate in the adipose tissue of the boar. Real-time PCR was used to quantify the expression of steroid sulfatase (STS) and several organic anion transporting polypeptides (OATPs) in the adipose tissue. Additionally, [^3^H]-androstenone sulfate was incubated with adipocytes or supernatant from homogenized fat to assess steroid uptake and conversion, respectively. A positive correlation existed between OATP-B expression and androstenone sulfate uptake (r = 0.86, *p* = 0.03), as well as between STS expression and androstenone sulfate conversion (r = 0.76, *p* < 0.001). Moreover, fat androstenone concentrations were positively correlated (r = 0.85, *p* < 0.001) with androstenone sulfate conversion and tended to increase with STS expression in early maturing boars. This suggests that androstenone sulfate uptake and deconjugation are mediated by OATP-B and STS, respectively, which may influence the development of boar taint in early maturing animals.

## 1. Introduction

Boar taint is an undesirable flavor or odor that develops in heated pork products from entire male pigs and is caused by the accumulation of androstenone (5α-androst-16-en-3-one) and skatole (3-methylindole) in the adipose tissue [1]. Androstenone is a sex pheromone that is synthesized in the testis during steroidogenesis and is sulfoconjugated by the sulfotransferase enzyme SULT2A1 before entering the systemic circulation [2,3]. Androstenone sulfate is the predominant form of androstenone in the peripheral plasma of the boar, accounting for approximately 70% of the total androstenone present in the circulation [3,4]. However, the role of androstenone sulfate in the boar has yet to be elucidated.

Sulfoconjugation involves the transfer of a sulfonate group (SO_3_^−^) from 3′-phosphoadenosine 5′-phosphosulfate (PAPS), a donor molecule, to the 3-hydroxyl position of an accepting steroid, which functions to inactivate and increase the water solubility of steroids [5]. Originally, sulfoconjugation was regarded as a mechanism to facilitate steroid excretion, and steroid sulfates were considered to be metabolic end products [5]. However, steroid sulfates were later found to function as steroid reservoirs, which are transported by membrane transporters belonging to the organic anion transporting polypeptide family (OATP) into various tissues and deconjugated by steroid sulfatase (STS) to return free bioactive steroids [6,7]. In humans, OATPs such as OATP-B, OATP-E, OATP-A, and OATP-D are encoded by the solute carrier organic anion (SLCO) gene and facilitate the sodium and ATP-independent uptake of sulfated steroids into several tissues [7,8], while STS is a microsomal enzyme that is expressed ubiquitously in small quantities, which hydrolyzes dehydroepiandrosterone sulfate (DHEAS) and estrone sulfate (E_1_S) [6].

The sulfoconjugation of androstenone is thought to require enolisation of the 3-keto group to produce a 3-enol intermediate, which can accept a sulfonate group from PAPS. Recently, a metabolite tentatively identified as androst-3-enol-3-sulfate was detected by liquid chromatography–mass spectrometry from Leydig cell culture and was found to return free androstenone, and not a hydroxylated metabolite, following chemical removal of the sulfate group [9]. Additionally, we have previously demonstrated that androstenone sulfate has a low binding capacity for porcine albumin, which is the carrier protein responsible for the transport of various steroids in the boar including free androstenone [10,11]. Consequently, androstenone sulfate circulates predominantly unbound in the porcine plasma and is presumably readily available for uptake into peripheral tissues [11,12]. On this basis, we hypothesized that androstenone sulfate may function as a steroid reservoir that is transported by OATPs into peripheral tissues such as the fat and hydrolyzed by STS to return free androstenone, which may subsequently accumulate to cause boar taint. Additionally, it is likely that this process could vary between individual animals due to differences in STS expression or hormonal status.

Therefore, the purpose of this study was to characterize the uptake and deconjugation of androstenone sulfate in the adipose tissue of boars with varying sulfatase expression and hormonal status to determine if androstenone sulfate can indirectly contribute to the development of boar taint.

## 2. Materials and Methods

### 2.1. Sample Collection

Plasma and backfat samples were obtained from 16 terminal cross [Duroc × (Yorkshire × Landrace)] boars. The boars were housed in pens with slatted floors in groups of approximately 5 beginning at 7 weeks of age and were provided ad libitum access to water and standard starter, grower, and finisher rations, formulated by Flordale Feed Mill Limited. All animals were used in accordance with the guidelines of the Canadian Council of Animal Care and the University of Guelph Animal Care Policy. A single pre-slaughter blood sample was collected from each animal at 120, 130, and 140 kg live weights. Plasma samples were analyzed using an E_1_S specific radioimmunoassay, previously described by Raeside and Renaud [13], in order to assess hormonal status. At 188 ± 3 days of age and approximately 160 kg live weight, the boars were electrically stunned and exsanguinated, and backfat samples were collected in liquid nitrogen from all boars and stored at −80 °C, allowing for subsequent evaluation of sulfatase activity and expression. Fresh backfat samples were also collected from 6 boars and immediately used for primary adipocyte culture. Fat androstenone concentrations were determined in backfat samples from all boars using an established reverse phase high-performance liquid chromatography (HPLC) technique previously described by Hansen-Møller [14], where dansylhydrazine is used to derivatize androstenone extracted from fat, which allows for subsequent quantification by florescence detection.

### 2.2. RNA Extraction and Gene Expression Analysis

Fat tissue was kept frozen in liquid nitrogen and pulverized with a mortar and pestle. Approximately 100 mg of powdered fat tissue was homogenized in 1 mL lysis buffer, and RNA was subsequently extracted using silica-based spin columns (RNeasy Lipid Tissue Mini Kit, Qiagen, Hilden, Germany). The RNA concentration was quantified using a NanoDrop 8000 spectrophotometer (Thermo Scientific, Waltham, MA, USA) and the RNA integrity was assessed using an Agilent 2000 Bioanalyzer (Agilent Technologies, Santa Clara, CA, USA).

RNA (1 µg) was reverse transcribed in 20 µL final volume with the High Capacity cDNA Reverse Transcription Kit (Applied Biosystems, Waltham, MA, USA) according to the manufacturer’s instructions. After incubation at 25 °C for 10 min, reverse transcription was carried out at 37 °C for 120 min, followed by 85 °C for 5 min. The resulting cDNA was diluted 5× and amplified by real-time PCR using a QuantaStudio Real Time PCR system (Thermo Fisher Scientific) in a 20 µL reaction volume containing 10 µL SsoAdvanced Universal Inhibitor-Tolerant SYBR Green Supermix (Bio-Rad, Hercules, CA, USA), 4.2 µL water, 5 µL diluted cDNA, and 200 nM of the respective primers, which are listed in Table 1. The run conditions were as follows: 3 min at 98 °C for polymerase activation, followed by 40 cycles of two-step qPCR (10 s of denaturation at 98 °C, 30 s of combined annealing/extension at 60 °C). Real-time PCR reactions were run in triplicate and gene expression was calculated using the ∆∆Ct method [15] using β-actin as a housekeeping gene and barrow fat as the calibrator.

### 2.3. Radiolabeled Androstenone Sulfate Synthesis and Purification

Human embryonic kidney (HEK293FT) cells purchased from ATCC (Manassas, VA. USA) were used to synthesize [^3^H]-androstenone sulfate as previously described by Bone and Squires [11]. Briefly, confluent HEK293FT cells were transfected with 6 µg/plate of the porcine sulfotransferase SULT2A1 (pSULT2A1) expression vector constructed as previously described by Laderoute et al. [2], and, after 48 h, treated with radiolabeled [^3^H]-androstenone (8 million CPM, 36 µCi/µmol, 0.1% ethanol). Cell culture media was collected 24 h later and analyzed by reverse-phase C18 HPLC using a Luna 5µ C18(2) HPLC column (250 × 4.60 mm) purchased from Phenomenex (Torrance, CA, USA). The 40 min HPLC profile used to confirm the conversion of free androstenone to androstenone sulfate by the HEK293FT cells was previously described by Laderoute et al. [9] and optimized for the elution of 16-androstene steroids. The elution of radiolabeled androstenone and androstenone sulfate occurred at approximately 32 and 16 min, respectively, and was monitored by a β-RAM model 2 isotope detector (IN/US Systems, Brandon, FL, USA). Radiolabeled androstenone sulfate was then isolated from the media by solid phase extraction using Sep-Pak C18 solid-phase chromatography cartridges (Waters, Milford, MA, USA), as previously described by Laderoute et al. [2]. The sulfated steroid fraction was dried under nitrogen and reconstituted in 100% ethanol.

### 2.4. Porcine Adipocyte Isolation

Mature adipocytes were isolated and cultured as previously described by Alexandersson et al. [23] with modifications. Briefly, 50 g of fat was minced, and the resulting homogenous mixture was added to 250 mL of digestion buffer (TC 199) containing 4.2 mM NaHCO_3_, 15 mM bovine serum albumin (BSA), 5.5 mM D-glucose, 1% penicillin–streptomycin, collagenase type I (1 mg/mL), 1.6 mM DNase, and 2.5 mM trypsin inhibitor. The fat was digested in a shaking water bath at 37 °C for approximately 40 min. The digested fat solution was then filtered using a 255 µm nylon mesh filter and transferred into a separation funnel. The remainder of the separation funnel was filled with warm wash buffer (TC 199) containing 4.2 mM NaHCO_3_, 15 mM BSA, 5.5 mM D-glucose, and 1% penicillin–streptomycin and then was gently inverted to mix the wash buffer and the digested fat solution. After 3 min, the fat layer had separated from the buffer, and the buffer was subsequently drained from the separation funnel. This was step was repeated three times to remove any remaining collagenase from the digestion. After washing, the mature adipocytes were collected into a 50 mL conical tube and centrifuged at 50× *g* for 8 min, which allowed oil from damaged cells and remaining wash buffer to be separated and removed.

### 2.5. Steroid Transport Studies using Mature Adipocytes

Isolated adipocytes (2 mL containing approximately 4 million cells) were suspended in 5 mL incubation buffer (DMEM/F12 mixed 1:1 (*v*/*v*) with α-MEM) containing 15.7 mM HEPES, 17.5 nM insulin, 1% fetal bovine serum (FBS), and 1% penicillin–streptomycin in 50 mL flasks and was incubated with [^3^H]-androstenone sulfate (24,000 CPM, 18.3 nCi/nmol) for 3 to 24 h in a shaking water bath at 37 °C with 95% air and 5% CO_2_. [^3^H]-Androstenone sulfate was incubated with media in the absence of isolated adipocytes as a negative control. All incubations were run in triplicate. Following incubation, the adipocytes were frozen, and the media was removed using a syringe. The adipocytes were then rinsed with wash buffer and melted in a hot water bath. Steroids were extracted once with 8 mL methanol, which was dried to a total volume of approximately 500 µL under nitrogen. The media was extracted twice with 4 mL ether, which was dried under nitrogen and reconstituted with 50% acetonitrile (500 µL). The media and fat extracts were then filtered and analyzed by reverse-phase C18 HPLC using the aforementioned 40 min HPLC profile optimized for the elution of 16-androstene steroids [9]. Steroid transport was calculated from the sum of free and sulfated androstenone present in the fat extract, which was quantified from the peak areas detected for each steroid and expressed as a percentage of the total steroid added to the incubation. This was used to calculate total steroid uptake (pmol) and steroid uptake rate (pmol/h).

### 2.6. Sulfatase Assay

The deconjugation of androstenone sulfate in the fat was quantified using a sulfatase assay previously described by Dalla Valle et al. [7] with modifications. Briefly, 2 g of backfat was thinly sliced and homogenized in 5 mL buffered medium (100 mM KCl, 16 mM K_2_HPO_4_, 4 mM KH_2_PO_4_, 1 mM DTT, 1 mM EDTA, 4 mM nicotinamide). The homogenate was centrifuged at 2000× *g* for 15 min at 4 °C, and 1 mL total volume of the resulting supernatant was incubated with [^3^H]-androstenone sulfate (28,000 CPM, 32 nCi/nmol) in a shaking water bath at 37 °C for 3 to 24 h. [^3^H]-Androstenone sulfate was incubated in buffer as a negative control. All incubations were run in triplicate.

Incubations were terminated and steroids were extracted twice with 2 mL ether, which was dried under nitrogen and reconstituted in 50% acetonitrile (300 µL). The extracted steroid solution was then filtered with a 0.2 µm nylon syringe filter (Fisher Scientific, Toronto, ON, Canada) and analyzed by reverse-phase C18 HPLC using the 40 min HPLC profile previously described. Steroid conversion was calculated from the percentage of free androstenone that was produced from androstenone sulfate, which was quantified from the respective peak area detected for each steroid. This was used to calculate the total conversion (pmol) and the conversion rate (pmol/h) of androstenone sulfate.

### 2.7. Statistical Analysis

Statistical analysis was conducted using SAS 9.4 (SAS Institute, Cary, NC, USA). Differences between animals in gene expression, fat androstenone concentrations, and the conversion of androstenone sulfate were evaluated using Student’s *t*-test with a significance level of *p* < 0.05. Additionally, Pearson correlation coefficients were determined for the following: (1) STS expression vs. OATP expression, (2) OATP expression vs. the rate of androstenone sulfate uptake into the fat, (3) STS expression vs. the percentage of androstenone sulfate converted to free androstenone, (4) STS expression vs. the concentration of androstenone in the fat. Correlations were calculated using the following model and considered statistically significant at *p* < 0.05:

$$\rho =\frac{\sigma_{xy}}{\sqrt{\sigma_{x}^{2}\sigma_{y}^{2}}}$$

where $\sigma_{x}^{2}$ is the variance of the *x* variable, $\sigma_{y}^{2}$ is the variance of the *y* variable, and *σ_xy_* is the covariance between *x* and *y*.

## 3. Results

### 3.1. Gene Expression Differences

**Figure 1:**
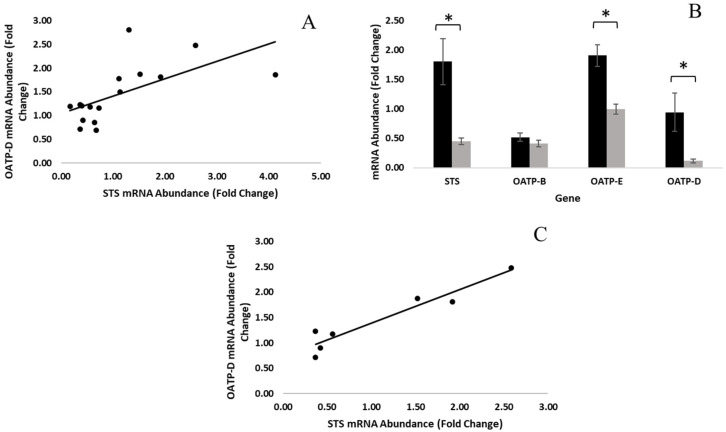
(**A**) The expression of STS in the fat was positively correlated (r = 0.63, *p* = 0.01) with the expression of OATP-D (*n* = 16). (**B**) The expression of STS, OATP-B, OATP-E, and OATP-D was compared between boars classified as high-sulfatase (black, *n* = 8) or low-sulfatase (grey, *n* = 8) animals. Data are expressed as the mean ± standard error, and significant differences (*p* < 0.05) are denoted by (*). (**C**) The expression of STS in the fat was positively correlated (r = 0.96, *p* < 0.01) with the expression of OATP-D in late maturing boars (*n* = 7).

RNA was extracted from fat tissue, and STS and OATP expression was quantified by real-time PCR to evaluate differences in gene expression between animals. All adipose tissue samples (*n* = 16) expressed STS, OATP-B, OATP-E, and OATP-D, while OATP-A was not detected. A moderate positive correlation (r = 0.63, *p* = 0.01) existed between the expression of sulfatase and OATP-D (Figure 1A), which was the most abundant membrane transporter expressed in the fat. Conversely, the expression of STS was not well correlated with that of OATP-E or OATP-B.

To evaluate the relationship between sulfatase expression and the expression of other genes in the fat, we arbitrarily classified boars as high (*n* = 8, STS expression ≥ 0.70) or low (*n* = 8, STS expression ≤ 0.67) sulfatase animals. The expression of STS and OATPs in high- and low-sulfatase boars is shown in Figure 1B. Adipose tissue from high-sulfatase boars expressed significantly greater quantities of STS (*p* = 0.004), OATP-D (*p* < 0.001), and OATP-E (*p* = 0.03) mRNA than adipose tissue from low sulfatase boars, while the expression of OATP-B was approximately equal.

Hormonal status was assessed by quantifying E_1_S concentrations in the plasma at 120, 130, and 140 live weights, and animals were classified as early or late maturing on the basis of the cutoff level for high E_1_S production that was previously described by Zamaratskia et al. [24]. Boars with plasma E_1_S concentrations greater than 15.7 ng/mL at 130 kg live weight or less were considered to have a hormonal status consistent with early maturation (*n* = 9), and animals with plasma E_1_S concentrations of 15.7 ng/mL or less at 130 kg live weight were classified as late maturing boars (*n* = 7). STS and OATP expression were compared between early and late maturing animals to assess the relationship between hormonal status and gene expression in the fat. There was no significant difference (*p* > 0.05) in the expression of STS or any of the OATPs between early and late maturing boars. However, a strong positive correlation (r = 0.96, *p* < 0.001) was observed between the expression of STS and OATP-D in late maturing boars (Figure 1C) and was not well correlated in early maturing boars.

### 3.2. Time Course Analysis of Steroid Uptake and Conversion

**Figure 2:**
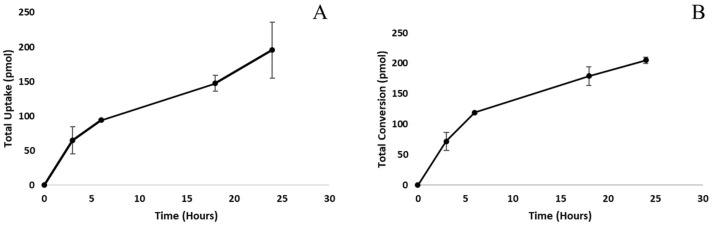
Isolated adipocytes were incubated with radiolabeled androstenone sulfate for 3, 6, 18, and 24 h. Steroids were extracted from the fat and detected by HPLC to quantify total uptake (**A**) or the production of free androstenone from androstenone sulfate (**B**). Data are presented as the mean ± standard error from 3 separate experiments.

Radiolabeled [^3^H]-androstenone sulfate was incubated with adipocytes (Figure 2A) or supernatant from homogenized fat tissue (Figure 2B) for 3, 6, 18, and 24 h to assess the time course for the uptake and conversion of androstenone sulfate by the adipose tissue, respectively. The uptake of androstenone sulfate increased over time and was greatest after 24 h (195.5 ± 40.4 pmol). Similarly, free androstenone production, resulting from the conversion of androstenone sulfate, increased significantly (*p* = 0.04) from 3 (71.7 ± 14.9 pmol) to 24 (205.2 ± 5.3 pmol) hours. The rate of both uptake and conversion was not linear but was greatest after 3 h (21.6 ± 6.6 pmol/h and 23.9 ± 14.9 pmol/h, respectively) and decreased over time, reaching 8.1 ± 1.7 pmol/h and 8.5 ± 5.3 pmol/h, respectively, after 24 h. Incubation times of 6 and 4 h were determined optimal for quantifying the uptake of androstenone sulfate and conversion of androstenone sulfate to free androstenone, respectively.

### 3.3. Uptake of Androstenone Sulfate by Adipocytes

**Figure 3:**
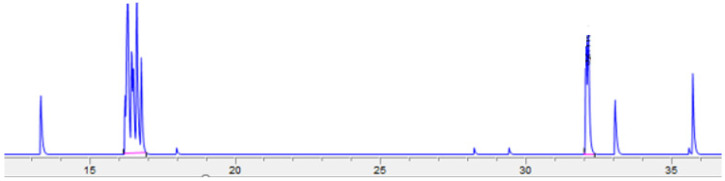
A chromatogram showing the production of radiolabeled free androstenone (32 min) from androstenone sulfate (16 min) following an incubation assessing steroid uptake and conversion by adipocytes.

**Figure 4:**
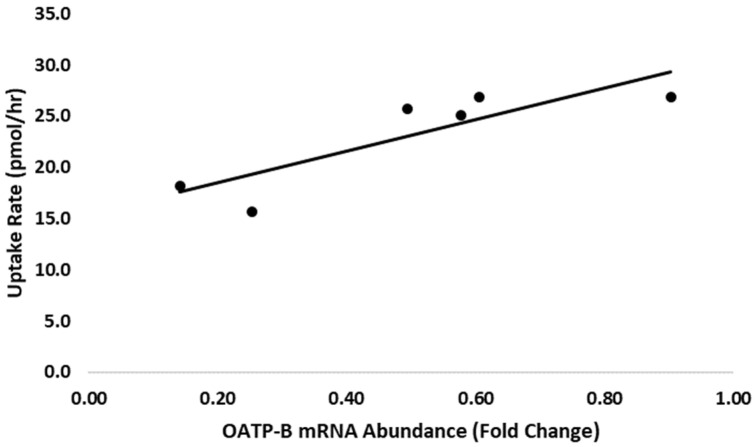
The expression of OATP-B in the fat was positively correlated (r = 0.86, *p* = 0.03) with the rate of uptake of androstenone sulfate by cultured adipocytes (*n* = 6).

Adipocytes isolated from fresh adipose tissue samples (*n* = 6) were incubated with [^3^H]-androstenone sulfate to characterize steroid transport, which was quantified by HPLC. A typical chromatogram depicting the uptake and subsequent conversion of androstenone sulfate to free androstenone is shown in Figure 3. The average uptake of androstenone sulfate by adipocytes over 6 h was 143.0 ± 12.0 pmol, or 23.1 ± 2.0 pmol/h. Additionally, 53.4 ± 10.1% of the androstenone sulfate transported into adipocytes was converted to free androstenone (76.4 ± 16.7 pmol). A strong positive correlation (r = 0.86, *p* = 0.03) was observed between the uptake of androstenone sulfate by adipocytes and the expression of the membrane transporter OATP-B (Figure 4), while the expression of OATP-D and OATP-E were not well correlated with androstenone sulfate uptake.

### 3.4. Conversion of Androstenone Sulfate to Free Androstenone

**Figure 5:**
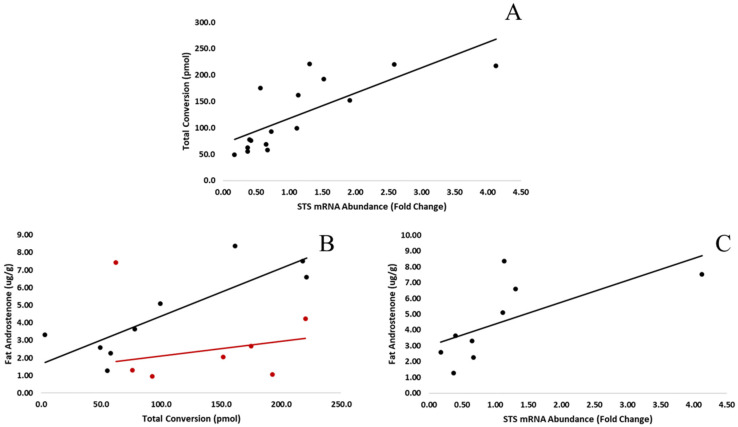
(**A**) The expression of STS in the fat was positively correlated (r = 0.76, *p* < 0.001) with the conversion of androstenone sulfate to free androstenone quantified by sulfatase assay (*n* = 16). (**B**) Fat androstenone concentrations were positively correlated (r = 0.85, *p* < 0.001) with the conversion of androstenone sulfate to free androstenone quantified by sulfatase assay in early maturing boars (black, *n* = 9) but not late maturing boars (red, *n* = 7). (**C**) A positive trend (r = 0.67, *p* = 0.05) was observed between the expression of STS in the fat and the concentration of androstenone quantified in the fat in early maturing boars (*n* = 9).

Fat tissue samples (*n* = 16) were homogenized in buffer, and the resulting supernatant was incubated with [^3^H]-androstenone sulfate and analyzed by HPLC to quantify the production of free androstenone from androstenone sulfate in the adipose tissue. The average expression of STS was 1.13 ± 0.26, and over 4 h incubations, the average production of free androstenone from androstenone sulfate was 123.7 ± 16.4 pmol, or 30.9 ± 4.1 pmol/h. Fat androstenone concentrations ranged from 0.96 to 8.38 µg/g with an average concentration of 3.77 ± 0.63 µg/g, and a strong positive correlation (r = 0.76, *p* < 0.001) was observed between the expression of STS and the production of free androstenone from androstenone sulfate (Figure 5A).

The relationship between fat androstenone concentration and androstenone sulfate conversion was examined in high and low sulfatase boars as well as early and late maturing animals to determine the effect of sulfatase expression and hormonal status on the deconjugation of androstenone sulfate in the fat. The average quantity of free androstenone produced from androstenone sulfate in boars with high sulfatase expression (*n* = 8) was 169.6 ± 18.6 pmol, which was significantly greater (*p* = 0.0015) than the steroid conversion quantified in low sulfatase boars (77.8 ± 14.3 pmol, *n* = 8). Additionally, fat androstenone concentrations were not significantly different (*p* > 0.05) between high (4.48 ± 1.03 µg/g) and low (3.06 ± 0.69 µg/g) sulfatase boars.

The production of free androstenone from androstenone sulfate was positively correlated (r = 0.85, *p* < 0.001) with fat androstenone concentrations in early maturing (*n* = 9) boars and was not well correlated in late (*n* = 7) maturing boars (Figure 5B). Additionally, fat androstenone concentrations in early maturing boars tended to increase with the expression of STS (r = 0.67, *p* = 0.05, Figure 5C); however, there were no significant differences (*p* > 0.05) in the conversion of androstenone sulfate or fat androstenone concentrations between early and late maturing boars.

## 4. Discussion

The accumulation of androstenone in the adipose tissue causes a meat quality issue in heated pork products from entire males, which is known as boar taint. In humans, the adipose tissue functions in an intracrine manner by supporting the uptake and conversion of DHEAS to free dehydroepiandrosterone (DHEA), which serves as a precursor for bioactive androgens and estrogens [7]. Therefore, in the present study, we investigated androstenone production from the conversion of androstenone sulfate in adipose tissue from boars with high and low sulfatase expression to examine the relationship between the deconjugation of androstenone sulfate and boar taint development.

OATPs facilitate the cellular uptake of substrates such as xenobiotics, bile acids, and steroid sulfates [25]. Following uptake, steroid sulfates such as E_1_S and DHEAS are deconjugated by STS to return estrone (E_1_) and DHEA, respectively [6]. Using real-time PCR, we established that porcine adipose tissue expressed STS and all membrane transporters except OATP-A, which is in concordance with the expression reported in human adipose tissue [7]. We identified a positive correlation between the expression of STS and OATP-D and determined that higher sulfatase expression was associated with greater expression of the membrane transporters OATP-D and OATP-E, but not OATP-B. In humans, E_1_S is a substrate of all three OATPs, while the cellular uptake of DHEAS is mediated by OATP-B and not OATP-D or OATP-E [25]. Boars express high levels of testicular sulfotransferases and often produce large quantities of E_1_S [26]. Plasma concentrations of E_1_S increase as boars reach sexual maturity [27,28], and concentrations of E_1_ in the fat of sexually mature boars are positively correlated with fat androstenone concentrations [24]. The positive correlation between the expression of STS and OATP-D in late maturing boars suggests that the uptake and deconjugation of E_1_S to produce bioactive E_1_ by the adipose tissue may be necessary to promote the onset of sexual maturity in late but not early maturing boars. Therefore, future research should investigate the uptake and deconjugation of E_1_S by the porcine adipose tissue to further characterize the effect of E_1_S production on sexual maturation and consequently the development of boar taint.

We identified a positive correlation between the uptake of androstenone sulfate by adipocytes and the expression of OATP-B, as well as the expression of STS and the conversion of androstenone sulfate to free androstenone. Additionally, boars that expressed higher levels of sulfatase converted significantly greater quantities of androstenone sulfate to free androstenone; however, the expression of OATP-B in boars with high and low sulfatase expression was approximately equal. These results suggest that the transport and deconjugation of androstenone sulfate is mediated by OATP-B and STS, respectively, and the quantity of androstenone sulfate that is converted to free androstenone in the adipose tissue depends on the expression of STS rather than OATP-B.

**Figure 6:**
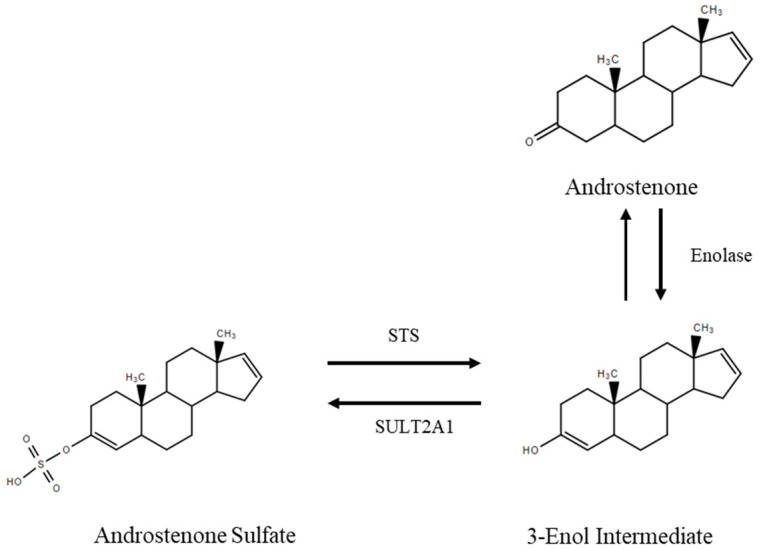
Proposed mechanism for the sulfoconjugation of androstenone and deconjugation of androstenone sulfate.

The STS-mediated hydrolysis of androstenone sulfate did not result in the production of a C_3_ hydroxysteroid, but rather returned the parent compound (androstenone), which is a C_3_ keto steroid. Consistent with our results, it has been previously reported that chemical hydrolysis of androstenone sulfate returns free androstenone and not a hydroxylated metabolite [9]. On the basis of the results of the present study, we predict that the STS-mediated hydrolysis of androstenone sulfate results in the production of the same 3-enol intermediate that is suspected to facilitate sulfoconjugation, with stabilization resulting in the movement of a double bond between C_3_ and C_4_ to the 3-keto position to produce free androstenone (Figure 6). This is consistent with the idea that androstenone sulfate functions as a steroid reservoir in the boar, which was originally proposed by Laderoute et al. [9]. However, future research is required to confirm the pathway mediating the deconjugation of androstenone sulfate. Furthermore, additional research should further investigate the relationship between OATP and STS expression and the uptake and deconjugation of androstenone sulfate in other breeds and with a larger number of animals.

Previous research has identified a positive correlation between testicular SULT2A1 expression and plasma concentrations of androstenone sulfate as well as a negative correlation between testicular SULT2A1 activity and fat androstenone concentrations [29]. On the basis of these results, researchers suggested that the sulfoconjugation of androstenone reduces boar taint development by decreasing the quantity of free androstenone available to accumulate in the fat [29]. However, the present study demonstrated that fat androstenone concentrations tended to increase with the expression of STS and were positively correlated with the production of free androstenone from androstenone sulfate in early but not late maturing boars. The development of boar taint depends on numerous factors that influence the rate of androstenone synthesis and metabolism and vary significantly between different breeds as well as individual boars within the same breed [30]. Our results suggest that the production of free androstenone from androstenone sulfate in the adipose tissue may have a significant impact on the development of boar taint in early maturing boars. Therefore, future research should further investigate the relationship between STS and fat androstenone concentrations in early maturing boars using larger sample sizes to determine if STS is a suitable candidate gene for boar taint.

## 5. Conclusions

This study demonstrated that the uptake and deconjugation of androstenone sulfate in the adipose tissue of the boar is facilitated by the membrane transporter OATP-B and STS, respectively. Additionally, we have shown that boars expressing higher levels of sulfatase have a greater expression of several OATPs and convert larger quantities of androstenone sulfate to free androstenone than boars with low sulfatase expression, which suggests that the uptake and deconjugation of steroid sulfates varies significantly between individual animals. Fat androstenone concentrations tended to increase with the expression of STS and were positively correlated with the production of free androstenone from androstenone sulfate in early maturing animals. These results suggest that the STS-mediated deconjugation of androstenone sulfate may be a significant cause of boar taint development in early maturing animals. Therefore, future research should further investigate this relationship to determine if STS is a suitable candidate gene for boar taint.

## Figures and Tables

**Table 1:** Primer sequences used in real-time PCR analyses.

Primer	Forward Sequence	Reverse Sequence	Tm (°C)	Reference
STS	5′-GAAGACAGGATCATTGACG-3′	5′-AGAACTTGGGTGTGAAGAAG-3′	85.7	[16,17]
β-Actin	5′-CGTGGACATCAGGAAGGAC-3′	5′-TCTGCTGGAAGGTGGACAG-3′	90.2	[17,18]
OATP-B	5′-TCAGCACACCCCTCTTCTTC-3′	5′-GACAAGGCGTGAGGTACTCC-3′	89.9	[19]
OATP-E	5′-TCGGGAAAACCATCAGAGAC-3′	5′-CCAGGTACCCGAACAAGGT-3′	90.8	[20]
OATP-D	5′-ATGTGCCTGTCTGGGAATCT-3′	5′-AGGTGCTGAGGTGTTTCCAT-3′	88.8	[21]
OATP-A	5′-TGCATTCAAACACCAGGAAA-3′	5′-GCATGTAATCCCACACCAAGA-3′	81.8	[22]

Primer sequences; annealing temperatures (Tm); and sequence references for steroid sulfatase (STS), β-actin, and organic anion transporting peptide (OATP) B, E, D, and A.
